# Plasma and breast milk pharmacokinetics of emtricitabine, tenofovir and lamivudine using dried blood and breast milk spots in nursing African mother–infant pairs

**DOI:** 10.1093/jac/dkx507

**Published:** 2018-01-04

**Authors:** Catriona Waitt, Adeniyi Olagunju, Shadia Nakalema, Isabella Kyohaire, Andrew Owen, Mohammed Lamorde, Saye Khoo

**Affiliations:** 1 Department of HIV Pharmacology, 70 Pembroke Place, University of Liverpool, Liverpool L69 3GF, UK; 2 Infectious Diseases Institute, Makerere University College of Health Sciences, Kampala, Uganda; 3 Royal Liverpool University Hospital, Prescot Street, Liverpool L7 8XP, UK; 4 Faculty of Pharmacy, Obafemi Awolowo University, Ife-Ife, Nigeria

## Abstract

**Background:**

Breast milk transfer of first-line ART from mother to infant is not fully understood.

**Objectives:**

To determine the concentrations of lamivudine, emtricitabine and tenofovir in maternal blood, breast milk and infant blood from breastfeeding mother–infant pairs.

**Methods:**

Intensive pharmacokinetic sampling of maternal dried blood spots (DBS), dried breast milk spots (DBMS) and infant DBS from 30 Ugandan and 29 Nigerian mothers receiving first-line ART and their infants was conducted. DBS and DBMS were collected pre-dose and at 5–6 timepoints up to 12 h following observed dosing. Infant DBS were sampled twice during this period. Lamivudine, emtricitabine and tenofovir were quantified using LC-MS/MS, with non-compartmental analysis to calculate key pharmacokinetic parameters.

**Results:**

Peak concentrations in breast milk from women taking lamivudine and emtricitabine occurred later than in plasma (4–8 h compared with 2 h for lamivudine and 2–4 h for emtricitabine). Consequently, the milk-to-plasma (M:P) ratio of lamivudine taken once daily was 0.95 (0.82–1.15) for AUC_0–12,_ whereas for AUC_12–20_ this was 3.04 (2.87–4.16). Lamivudine was detectable in 36% (14/39) of infants [median 17.7 (16.3–22.7) ng/mL]. For 200 mg of emtricitabine once daily, the median M:P ratio was 3.01 (2.06–3.38). Three infants (19%) had measurable emtricitabine [median 18.5 (17.6–20.8) ng/mL]. For 300 mg of tenofovir once daily, the median M:P ratio was 0.015 (0–0.03) and no infant had measurable tenofovir concentrations.

**Conclusions:**

Emtricitabine and lamivudine accumulate in breast milk and were detected in breastfeeding infants. In contrast, tenofovir penetrates the breast milk to a small degree, but is undetectable in breastfeeding infants.

## Introduction

In 2015, an estimated 1.4 million HIV-positive women became pregnant, of whom approximately 1 million received combination ART.^1^ Given that breastfeeding remains the only acceptable, feasible, affordable, sustainable and safe infant feeding option in low- and middle-income countries,^2^ the number of infants exposed to antiretroviral drugs through pregnancy and breastfeeding will continue to increase as countries adopt WHO guidelines for universal, lifelong access to ART.^3^

In most countries where universal breastfeeding is recommended, first-line ART comprises the NNRTI efavirenz, together with the NRTI tenofovir disoproxil fumarate and either lamivudine or emtricitabine in fixed-dose combination (FDC). It is important to understand the transfer of these drugs from mother to breastfed infant, since it has been demonstrated that low infant concentrations of drugs predispose them to the selection of HIV drug resistance should HIV transmission occur,^4^^,^^5^ and there continue to be conflicting safety data, for example regarding the safety of tenofovir on developing bone.^6^ The pharmacokinetic (PK) profiles in paired maternal and infant plasma have been reported for efavirenz^7^ and lamivudine,^8^ but only three studies have sought to measure tenofovir^9–11^ and a single study measured emtricitabine in the breast milk of HIV-positive mothers.^9^ These studies of tenofovir and emtricitabine did not conduct intensive PK sampling or present paired mother and infant data for analysis.

This study aimed to describe the PK transfer of the current WHO-recommended NRTI backbone of first-line ART regimens from mother to breastfed infant. Intensive PK data on these drugs in dried blood spots (DBS) and dried breast milk spots (DBMS) from Ugandan and Nigerian mother–infant pairs are reported.

## Methods

### Study design

#### Study sites and participants

Thirty Ugandan mother–infant pairs were recruited from the Infectious Diseases Institute, Makerere University, Kampala, Uganda. Table  1 summarizes the breakdown of the number of participants on each ART regimen. In summary, 30 mother–infant pairs contributed to the lamivudine and 18 to the tenofovir analyses. The study protocol and the material transfer agreement were approved by the University of Liverpool Research Ethics Committee, UK and the Joint Clinical Research Centre IRB, Uganda and the Uganda National Council for Science and Technology (HS1675).

**Table 1.** Patient characteristics

	Uganda (*n *=* *30)	Nigeria (*n *=* *29)
Regimen		
NVP twice daily+ZDV/3TC twice daily	9	1
NVP twice daily+TDF/3TC once daily	3	6
NVP twice daily+TDF/FTC once daily	0	7
EFV/TDF/3TC once daily	18	1
EFV/TDF/FTC once daily	0	13
EFV once daily+ZDV/3TC twice daily	0	1
Key patient characteristics		
maternal age (years), mean (range)	30 (21–37)	30 (20–38)
maternal weight (kg), mean (range)	61 (43–87)	59 (46–79)
CD4 count (cells/mm^3^), median (IQR)	527 (345–608)	689 (552–938)
infant age (days), mean (range)	101 (81–146)	143 (80–215)
infant weight (kg), mean (range)	5.9 (4–6.8)	6.2 (3–10)

3TC, lamivudine; TDF, tenofovir; FTC, emtricitabine; EFV, efavirenz; ZDV, zidovudine; NVP, nevirapine.

Twenty-nine mother–infant pairs were recruited from two hospitals in Benue State, Nigeria: Bishop Murray Medical Centre, Makurdi and St Monica’s Hospital, Adikpo. The ART regimens of each participant are summarized in Table  1, with 9 mother–infant pairs contributing to the lamivudine analysis, 27 to the tenofovir analysis and 16 to the emtricitabine analysis. Ethical approval was obtained from the National Health Research and Ethics Committee (NHREC), Abuja and Obafemi Awolowo University Teaching Hospitals Ethics and Research Committee, Ife-Ife, Nigeria. This cohort has previously been described in detail.^12–14^

In both cohorts, most infants were under 6 months of age and reportedly still being exclusively breastfed. All infants were fed on demand during the study to reflect real-life situations, with the time of the most recent breastfeeding being recorded.

#### Sampling scheme

Participants who were sampled prior to and at intervals after dosing were provided with a standard local meal followed by observed dosing of their usual medication. For patients who were sampled at later timepoints following a self-administered dose, no dietary restrictions were applied, in keeping with standard practice.

Participants receiving 150 mg of lamivudine twice daily had maternal DBS (mDBS) and DBMS sampling prior to dosing and at 1, 2, 4, 6 and 8 h post-dose (Uganda, *n *=* *9) or prior to dosing and at 0.5, 1, 2, 4 and 8 h post-dose (Nigeria, *n *=* *2). Participants receiving 300 mg of lamivudine once daily had mDBS and DBMS sampling prior to dosing and at 0.5, 1, 2, 4 and 8 h post-dose (Nigeria, *n *=* *7), prior to dosing and at 1, 2, 4, 6 and 8 h post-dose (Uganda, *n *=* *3) or at 12, 16 and 20 h post-dose (Uganda, *n *=* *18).

All participants receiving 200 mg of emtricitabine once daily were at the Nigerian sites and had mDBS and DBMS sampling prior to dosing and at 0.5, 1, 2, 4, 8 and 12 h post-dose.

Participants receiving 300 mg of tenofovir disoproxil fumarate once daily had mDBS and DBMS sampling performed prior to dosing and at 0.5, 1, 2, 4, 8 and 12 h post-dose (Nigeria, *n *=* *27), prior to dosing and at 1, 2, 4, 6 and 8 h post-dose (Uganda, *n *=* *3) or at 12, 16 and 20 h post-dose (Uganda, *n *=* *18).

For all analytes, infant DBS samples were collected at 2 and 8 h post-dose and, for the later timepoints sampled in Uganda, at 14 and 20 h post-dose.

#### Sampling methods

In Uganda, mDBS samples were prepared by accurately spotting 50 μL of blood onto the Whatman 903 Protein Saver card and plasma was harvested from the remaining blood. DBS samples were collected from infants, after sterile skin cleaning and heel prick, using a 2 mm safety lancet (BD, Oxford, Oxfordshire, UK). All samples were stored locally at −80 °C, transported to the Department of Molecular and Clinical Pharmacology, University of Liverpool, UK on dry ice and then kept at −80 °C prior to analysis.

In Nigeria, whole blood samples from both mother and infant were collected as DBS samples after sterile skin cleaning and finger prick using a 2 mm safety lancet (BD, Oxford, Oxfordshire, UK). The first drop of blood was discarded and subsequent blood drops were collected on the sample collection areas of Whatman 903 cards. Plasma was not harvested at this site owing to logistical constraints.

At both sites, within 2 min of each DBS collection, about 5 mL of breast milk was manually expressed by the mother and 30 μL aliquots were spotted onto each circle on the Whatman 903 Protein Saver card.

### Drug quantification and analysis

#### DBS and DBMS

Lamivudine, emtricitabine and tenofovir were quantified using LC-MS/MS using a method recently described.^15^ In brief, the entire DBS or DBMS was removed using a 12 mm punch. Initial extraction of the DBS was with 0.1% formic acid in water for 5 min prior to the addition of deuterated internal standard. 800 μL of acetonitrile was then added prior to centrifugation, evaporation and reconstitution in 100 μL of water/acetonitrile (99/1, v/v). DBMS samples were extracted with 1 mL of acetonitrile/water (70/30, v/v) by tumbling for 30 min in the presence of internal standard, with evaporation and reconstitution as for DBS. A reverse-phase Phenomenex column was used on an HPLC connected to a TSQ Quantum Ultra triple quadrupole mass spectrometer (Thermo Electron Corporation, Hemel Hempstead, Hertfordshire, UK) equipped with a heated electrospray ionization source. Xcalibur Software and LCquan (version 2.6.1, Thermo Fisher Scientific, Hemel Hempstead, Hertfordshire, UK) were used for method set up, data acquisition, data processing and reporting.

The assay was shown to yield consistent data across a range of haematocrits down to 22%. Cross-validation of lamivudine and tenofovir concentrations was undertaken by analysis of paired maternal plasma and DBS obtained from the same blood draw. The lower limit of quantification in DBS was 16.6 ng/mL for all analytes and in breast milk was 16.6 ng/mL for lamivudine and emtricitabine and 4.2 ng/mL for tenofovir.

#### Plasma

Lamivudine and tenofovir were quantitated from 100 μL of plasma. 20 μL of internal standard mixture, lamivudine-IS (2.5 μg/mL) and emtricitabine-IS (2.5 μg/mL) was added. 400 μL of acetonitrile was added to precipitate the proteins followed by centrifugation at 4000 rpm for 10 min. 300 μL of the above supernatant was removed and evaporated to dryness under a nitrogen steam. The dried residue was dissolved in 100 μL of water/acetonitrile (99/1, v/v) and transferred into auto sampler vials. The samples were analysed using a validated in-house LC-MS/MS method. Chromatographic and mass spectrometer conditions were the same as detailed above for DBS and DBMS analysis. For plasma analysis, the calibration curve was linear from 5 to 5000 ng/mL for all analytes, with a lower limit of quantification of 5 ng/mL.

### Statistical methods

Non-compartmental analysis of PK data to obtain *C*_max_, *T*_max_ and AUC was undertaken using data from both sites using WinNonLin (Phoenix, version 6.1; Pharsight Corp., Mountain View, CA, USA).

To evaluate the agreement between mDBS and plasma concentrations of the drugs, the paired DBS and plasma samples from Ugandan mothers were correlated using linear regression. A correction factor was obtained to standardize mDBS concentrations to an estimated plasma value. This and Bland–Altman analysis were undertaken using GraphPad Prism (version 5.00 for Windows, GraphPad Software, San Diego, CA, USA).

Inadequacy of low-temperature storage facilities in remote study locations in Nigeria precluded the collection of plasma samples from the Nigerian cohort who were receiving emtricitabine-containing ART regimens. Therefore, the correlation between mDBS and plasma for tenofovir in this study was compared with that reported by Zheng *et al.*^16^ who reported good correlation between plasma and DBS for both tenofovir and emtricitabine. If results were similar for tenofovir, a previously published correction factor for emtricitabine was used to estimate plasma concentrations from the DBS data.

Since the M:P ratio varies over the dosing interval owing to a number of factors, including delayed peak in breast milk concentration resulting from slower distribution into the breast compartment,^8^^,^^17^ the ratio of the AUC for paired DBMS and estimated plasma was calculated arithmetically to summarize the relationship between drug exposure in the breast milk compared with the plasma over the entire dosing interval. This is presented as the M:P AUC ratio.

To estimate clinical relevance, the breast milk concentrations were interpreted as the percentage of the recommended infant dose that would be ingested by an exclusively breastfed infant. Tenofovir is not recommended for children under the age of 2 years and therefore this calculation was not performed for tenofovir concentrations. The standard assumption of 150 mL/kg/day milk intake was made.

## Results

### Patient populations

Ugandan and Nigerian populations were similar in terms of maternal age, weight and CD4 count and infant age and weight (Table  1). All women had started receiving ART prior to or during their recent pregnancy and were therefore all at steady-state by the time of sampling. All Ugandan and 80% of Nigerian women stated they were exclusively breastfeeding at the time of the study.

### Cross-validation between mDBS and plasma

**Figure 1.**
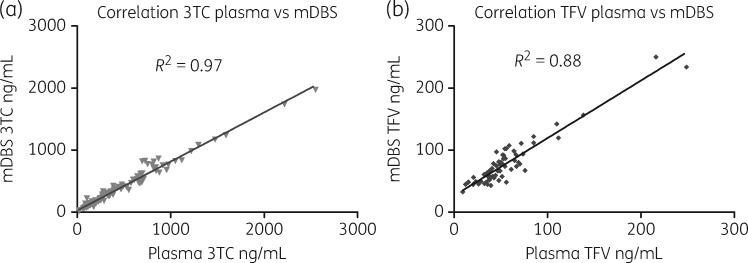
Correlation plots for concentration of lamivudine (a) and tenofovir (b) from maternal plasma and mDBS. 3TC, lamivudine; TFV, tenofovir.

Plasma and mDBS concentrations of lamivudine and tenofovir showed a strong positive correlation (*R*^2^* *=* *0.97 and 0.88, respectively). Using a correction factor derived from the average plasma: mDBS ratio (*y *=* *0.88*x* for lamivudine and *y *=* *1.57*x* for tenofovir), Bland–Altman analysis indicated good agreement between the two methods (Figure 1), noting slightly lower agreement between the plasma and mDBS analyses at higher analyte concentrations for lamivudine and tenofovir. However, it should be noted that the majority of data points were at concentrations that demonstrated good agreement.

This approach for standardizing by a correction factor is similar to that employed by Zheng *et al.*^16^ in the development of a DBS assay for tenofovir and emtricitabine. Our study yielded a similar correction factor for tenofovir, but we were unable to compare plasma and DBS concentrations for emtricitabine. Therefore we used the correction factor of *y *=* *0.8*x* as derived by Zheng *et al.*^16^ to standardize emtricitabine DBS data for plasma. Concentration–time profiles for each drug were obtained using the estimated plasma and breast milk concentrations.

### PK data

**Figure 2.**
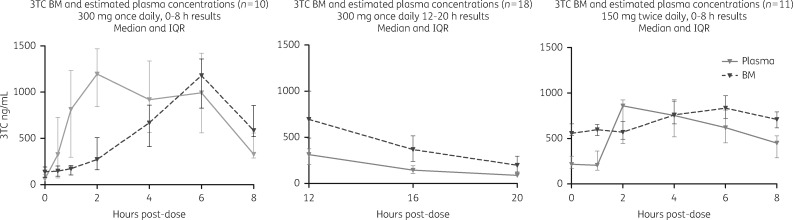
PK profiles of lamivudine in breast milk and estimated plasma. 3TC, lamivudine; BM, breast milk.

**Figure 3.**
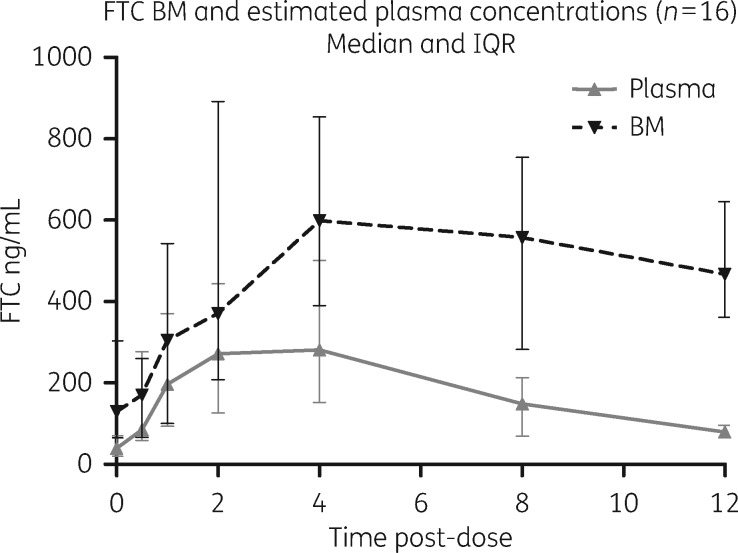
PK profiles of emtricitabine in breast milk and estimated plasma. FTC, emtricitabine; BM, breast milk.

**Figure 4.**
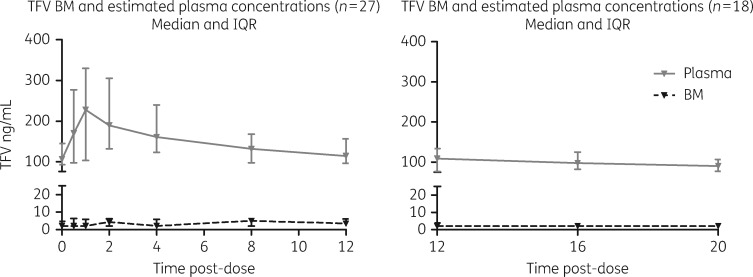
PK profiles of tenofovir in breast milk and estimated plasma. TFV, tenofovir; BM, breast milk.

The key PK parameters (*C*_max_, *T*_max_ and AUC and the M:P ratio) for all three analytes in plasma (estimated) and breast milk are presented in Table  2 and summarized in Figures 2–4. Using AUC as a summary measure of drug exposure, the findings for each drug are summarized below.

**Table 2.** Summary of PK parameters

Drug	Dose	Estimated plasma			DBMS			AUC_0–12_ M:P ratio
		*T*_max_ (h)	*C*_max_ (ng/mL)	AUC_0–12_ (ng·h/mL)	*T*_max_ (h)	*C*_max_ (ng/mL)	AUC_0–12_ (ng·h/mL)	
FTC	200 mg	2 (0.5–4)	384 (269–530)	2371 (1276–3344)	4 (2–8)	843 (702–1132)	4991 (4094–7179)	3.01 (2.06–3.38)
3TC^a^	150 mg twice daily	4 (2–4)	674 (637–715)	3644 (2817–3783)	6 (4–6)	908 (772–1015)	5937 (5263–6130)	1.65 (1.59–1.92)
3TC	300 mg once daily	2 (1.5–3)	915 (635–1118)	4541 (3160–5997)	6 (4–8)	663 (445–890)	4420 (2106–5502)	0.95 (0.82–1.15)
TFV	300 mg once daily	1 (0.5–2)	293 (176–391)	1591 (1193–2234)	4 (1–6)	5.98 (0–8.05)	21.1 (0–36.2)	0.015 (0–0.03)

FTC, emtricitabine; 3TC, lamivudine; TFV, tenofovir.

All results are presented as median (IQR).

a For 3TC 150 mg twice daily, the AUC presented is 0–8 h, not 0–12 h.

#### Lamivudine

Of the 39 women who received lamivudine-containing regimens, 28 took 300 mg once daily and 11 took 150 mg twice daily. Of these, 10 were administered their medication in the morning as part of a nevirapine-based regimen (and therefore had intensive PK from 0 to 8 h post-dose) and 18 women on efavirenz-based regimens took their medication in the evening, allowing sampling from 12 to 20 h post-dose. PK profiles are illustrated in Figure 2. For women taking regimens containing 150 mg twice daily, the estimated plasma AUC_0–8_ was 3644 (2817–3783) ng·h/mL and the breast milk AUC_0–8_ was 5937 (5263–6130) ng·h/mL, giving a median AUC_0–8 _M:P ratio of 1.65 (1.59–1.92). Among women taking 300 mg once daily, as part of an FDC, the estimated plasma AUC_0–12_ was 4541 (3160–5997) ng·h/mL and the breast milk AUC_0–12_ was 4420 (2106–5502) ng·h/mL, giving an AUC_0–12 _M:P ratio of 0.95 (0.82–1.15). Women who took 300 mg once daily and were sampled from 12 to 20 h after dose had an estimated plasma AUC_12–20_ of 2510 (1803–3071) ng·h/mL, a breast milk AUC_12–20_ of 7600 (4913–10 810) ng·h/mL and an AUC_12–20 _M:P ratio of 3.04 (2.87–4.16).

Lamivudine was detectable in the DBS of 36% (14/39) of infants, at a median of 17.7 (16.3–22.7) ng/mL. Of these, detectable levels were found in 3/11 (27%) infants whose mothers were exposed to twice-daily dosing, 7/10 (70%) infants whose mothers had 300 mg dosing that morning and 4/18 (22%) of those whose mothers had dosed the previous evening.

#### Emtricitabine

Data were available from 16 mothers taking 200 mg of emtricitabine once daily, as part of an FDC, with PK profiles in estimated plasma and DBMS illustrated in Figure 3. The estimated plasma AUC_0–12_ was 2371 (1276–3344) ng·h/mL, the breast milk AUC_0–12_ was 4991 (4094–7179) ng·h/mL and the median AUC_0–12 _M:P ratio was 3.01 (2.06–3.38).

Three infants (19%) had measurable emtricitabine concentrations, at 17.5, 18.8 and 19.4 ng/mL.

#### Tenofovir

Forty-eight mothers received tenofovir-containing regimens, all with once-daily dosing of 300 mg. Of these, 27 received their medication in the morning enabling PK sampling from 0 to 12 h and 18 took their dose in the evening allowing measurement of PK from 12 to 20 h post-dose. Three Ugandan mothers had sampling from 0 to 8 h post-dose; given the small size of this group, these women were excluded from the following analysis. PK parameters are illustrated in Figure 4.

The estimated plasma AUC_0–12_ was 1591 (1193–2234) ng·h/mL, the breast milk AUC_0–12_ was 21.1 (0–36.2) ng·h/mL and the median AUC_0–12 _M:P ratio was 0.015 (0–0.03). The estimated plasma AUC_12–20_ was 1413 (1144–1830) ng·h/mL and the breast milk AUC_12–20_ was 0 (0–37.2) ng·h/mL, giving a median AUC_12–20 _M:P ratio of 0 (0–0.02).

No infant had a measurable tenofovir concentration.

## Discussion

Full PK profiles of lamivudine, emtricitabine and tenofovir in plasma (DBS-derived) and breast milk across the dosing interval are presented in addition to infant DBS concentrations. Lamivudine and emtricitabine both penetrate breast milk and although the relative ‘dose’ ingested by the infant was less than one percent of the recommended treatment dose for infants of similar weight, these drugs are detectable in a proportion of infants.

It is notable that the absorption of lamivudine into the breast milk lags behind that of plasma, with a time to maximum concentration of 6 h compared with 2 h, respectively. It is then eliminated more slowly from breast milk than blood. Taken together, this means that the milk-to-plasma ratio increased from a median of 0.95 between 0 and 12 h to a median of 3.04 from 12 to 20 h after dose. This has previously been noted in a study investigating the PK profile of lamivudine in blood and breast milk, which also reported similar AUCs for both maternal plasma and breast milk.^8^ Similarly, a recent study investigating blood and breast milk emtricitabine concentrations among women taking emtricitabine/tenofovir as HIV prevention found an M:P ratio of 0.63 and of 2.1 at 1–2 and 23–24 h post-dose, respectively,^18^ suggesting the differential PK of emtricitabine may follow a similar pattern; that 96% of infants reportedly had detectable emtricitabine at a median of 13.2 ng/mL indicates considerable exposure. In that study, although authors comment on ‘less variability’ in emtricitabine concentrations in breast milk compared with plasma throughout the dosing interval, the selected timepoints suggest that the study design may have missed the peak breast milk concentration. Our findings from undertaking intensive PK sampling in paired mDBS and DBMS suggest caution when interpreting M:P values from single or few timepoints as this may result in misleading conclusions. For example, consideration of early timepoints alone might result in underestimation of the total exposure of the breastfed infant to the drug in question.

It is important to understand the clinical consequences of infant exposure to low concentrations of lamivudine and emtricitabine. Evidence regarding low-level drug exposure and emergence of HIV resistance drawn from the Kisumu Breastfeeding Study (KiBS)^5^ and Breastfeeding, Antiretrovirals and Nutrition Study (BAN)^4^ indicated NRTI resistance in cases in which prevention of mother-to-child transmission (PMTCT) failed to prevent infant infection. However, these studies involved ART regimens that are no longer recommended as first-line therapy during pregnancy and breastfeeding and took place in an era before universal lifelong ART for this population was recommended. It is notable that whilst integrase strand transfer inhibitors are increasingly recommended in place of NNRTI in first-line regimens, and are being adopted in low- and middle-income countries, the NRTI backbone will remain a key component. Larger longitudinal cohort studies including infant PK and clinical follow-up will be required to fully explore this question.

The finding of low tenofovir in breast milk is not unexpected since tenofovir is a dianion at physiological pH and suffers from poor membrane permeability; this is consistent with previous work.^9–11^^,^^18^ Furthermore, tenofovir has poor oral bioavailability, hence it is administered as the pro-drug tenofovir disoproxil fumarate or increasingly as the phosphonoamidate pro-drug tenofovir alafenamide. Therefore the finding that infants exposed to low concentrations of tenofovir in breast milk have undetectable tenofovir concentrations would be expected. However, this contrasts with a study undertaken in Malawi, assessing mothers and infants at 1 and 12 months post-partum in which low concentrations of tenofovir [median (IQR) 24 (0–51.6) and 0 (0–29.9) ng/mL] were measurable at 1 and 12 months, respectively, although breast milk concentrations were low with an M:P ratio of less than 0.1.^11^

The tables and figures present the summary data for the study population, expressed as median and IQR. However, considerable inter-individual variability was seen. Current work seeks to delineate the covariates influencing this and the clinical consequences thereof. Changes to WHO recommendations which will impact pregnant and breastfeeding women will need to be evaluated in suitable populations. Examples include the ongoing DolPHIN-1 study (NCT02245022) evaluating dolutegravir exposure in pregnant mothers with HIV and their neonates and NCT02499874, evaluating 400 mg of efavirenz in pregnant women. Breast milk elimination of tenofovir alafenamide has not yet been reported.

### Limitations

Due to the logistical challenges of undertaking intensive PK sampling in post-partum mothers and their infants, we were unable to perform a full 24 h profile. However, by enrolling participants with similar characteristics for early (0–12 h) and late (12–20 h) sampling, we were able to consider the full dosing interval.

Ninety percent of mother–infant pairs were enrolled when the infant was less than 6 months of age, and it was stated to the investigators that they were exclusively breastfeeding their infants, in accordance with WHO and national policies. However, it was not possible to verify this with certainty and it is possible that some of the study participants may have introduced mixed feeding. Although this may have resulted in lower infant concentrations of drugs due to a lower volume of ingested milk, it is unlikely to have impacted the maternal plasma and breast milk PK profiles.

It was infeasible to harvest plasma from the Nigerian participants to enable direct comparison of mDBS and plasma concentrations for emtricitabine; hence we used a previously published correction factor to standardize between the two matrices. However, such analysis was undertaken for lamivudine and tenofovir among the Ugandan cohort, yielding tenofovir results comparable with published data.

### Conclusions

Lamivudine and emtricitabine accumulate in breast milk with M:P ratios ranging from 0.82 to 4.16 and 2.06 to 3.38, respectively, over the dosing interval, resulting in detectable concentrations in a high proportion of infants. Tenofovir is detectable at low concentrations in breast milk, but was not measurable in any of the 47 infants sampled.
